# Prevalence and correlates of unintended pregnancy in Ghana: Analysis of 2014 Ghana Demographic and Health Survey

**DOI:** 10.1186/s40748-018-0085-1

**Published:** 2018-09-05

**Authors:** Edward Kwabena Ameyaw

**Affiliations:** Department of Population and Health, University of Cape Coast, Cape Coast, Ghana

**Keywords:** Unintended pregnancy, Women, Prevalence, Ghana, Correlates

## Abstract

**Background:**

Unintended pregnancies increase levels of stress, adoption of risky behaviours and impact on women’s general quality of life. In Ghana, in spite of the paucity of literature on unintended pregnancies, the phenomenon is high especially among women in the early years of their reproductive health. This study therefore sought to investigate the prevalence and correlates of unintended pregnancies in Ghana.

**Methods:**

This study made use of data from the 2014 Ghana Demographic and Health Survey. Descriptive statistics were conducted whereby prevalence of unintended pregnancy was presented in proportions. This was followed by binary logistic regression to investigate correlates associated with unintended pregnancy.

**Results:**

Descriptively, unintended pregnancies were high among women aged 15–19 years (69.4%), unmarried women (45.1%) and non-working women (40.0%). Factors found to be associated with the phenomenon were age, parity and level of education. The binary logistic regression revealed that women in middle wealth category were 1.42 times more probable of having unintended pregnancy than poor women whilst rich women were less likely to experience unintended pregnancy [OR = 0.89, CI = 0.35–0.79] as compared to poor women. Again, urban women were more likely to experience unintended pregnancies as compared rural women [OR = 1.39, CI = 0.86–1.95].

**Conclusion:**

The study has indicated that specific interventions must be targeted at different categories of women. The Reproductive and Child Health unit of the Ghana Health Service ought to collaborate with non-governmental organisations to intensify access to well-tailored family planning services among adolescents and young women, women out of marriage and the non-working category.

## Background

The imminent birth of a child occurs within a complex social context with associated implications for the mother, expected child, the family as well as the society at large. When pregnancy takes place unintendedly, the associated complications are more probable to be enormous [1]. Unintended pregnancies are pregnancies that are either mistimed (wanted earlier or later than occurred) or unwanted (not wanted at all) [2]. As of 2008, 75 million unintended pregnancies occurred among women in LMICs with 23% of these occurring in Sub-Saharan Africa [3, 4]. Unintended pregnancies although declining, has been substantially high as it stood at 213 million on the global scale in 2012 [1].

Unintended pregnancies sometimes increase levels of stress, adoption of risky behaviours, delayed initiation of prenatal care, unwillingness to seek social support during pregnancy, impacts on women’s quality of life in general and threaten households’ economic status [5–8]. In spite of international commitment toward improving maternal and child health over the past decades, health implications of unintended pregnancies constitute a major public health concern, especially for women living in developing countries [9, 10]. As noted by the ecosocial theory, pregnancy intentions encompass affective, cognitive, cultural and contextual dimensions. The ecosocial theory further holds that *s*ocial issues such as one’s economic standing and marital status influence health related outcomes in general [11, 12]*.*

As of 2014, the Ghana Demographic and Health Survey indicated that unintended pregnancy stands at 29.8% signifying a relative decline from what had initially been reported [13]. Meanwhile, the phenomenon is still high among some sub-categories, for instance it stands at 69% and 42.8% among women aged 15–19 and 20–24 years respectively. Unintended pregnancies in Ghana and other sub-Saharan Africa might imply limited access to reproductive health services among women in these geographical areas. This manifests in limited access to family planning services, low reproductive health rights and truncated empowerment of women [14]. In order to reverse the prevalence of unintended pregnancies, associated factors are to be explored and understood in order to inform suitable interventions. The current study digresses from what has been explored and contributes to the body of knowledge by investigating national prevalence of unintended pregnancies and factors inducing this phenomenon by dwelling on data from the 2014 Ghana Demographic and Health Survey (GDHS).

## Methods

### Source of data

The study used women’s file from the 2014 Ghana Demographic and Health Survey (2014 GDHS) [13]. The GDHS is a national survey carried out every five years. The survey is conducted by the Ghana Statistical Service and Ghana Health Service with technical support from the ICF International. It principally focuses on maternal and child health conditions among developing countries and as such was adjudged suitable for this study. It specifically covers issues such as unintended pregnancy, family planning, nutrition, fertility, health insurance, prenatal care, choice of place of delivery and postnatal care [13]. The 2014 edition interviewed 9396 women aged 15–49 years from 11,835 households emerging from 427 clusters nationwide. However, 4294 women constituted the sample for this study. The inclusion criteria for this sample was that a woman ought to have birth history within the five year period preceding the survey and must also fall within ages 15 and 49. Considering the prime focus of the survey to generate recent and reliable information on fertility, family planning and related issues, five year period is considered suitable to achieve this objective. The survey had a response rate of 97% and it was conducted with an updated frame from the 2010 Population and Housing Census (PHC). The dataset was requested from Measure DHS website. Access to the dataset was granted following approval of the request to use the data [13].

### Description of variables

The dependent variable was unintended pregnancy. During the survey, respondents were asked “When you got pregnant, did you want to get pregnant at that time?” The responses to this question were either “Yes” = 1 or “No” = 2. In all, ten independent variables were selected and these independent variables were not chosen arbitrary but based on two main reasons. First, the choice of these variables was premised on the proposition of the underlying theory guiding this study (ecosocial theory) which posits that some societal structures (such as religious affiliation, marital status and educational attainment) induce women’s perception about intendedness of pregnancies and secondly by varied conclusions by some previous studies [14, 15]. Following this, the ten independent variables selected were age, wealth status, educational level, religion, marital status, residential status, occupation, parity, contraceptive use and intention as well as decision maker for contraceptive use. Some of these variables were re-coded to fit the purpose of the study. These were wealth status re-coded as poor = 0, middle = 1, rich = 2 whereby women in the least wealth category were referred to as the “poor”; those who are above the least wealth category but not up to those in the high category were categorised as middle whilst those in the high wealth category were considered as rich. Parity was recoded as “between one (1) and four (4)”=0 and “more than four (4+)”=1 taking into cognisance Ghana’s current total fertility rate of 4.2 [13]. With respect to occupation, someone was considered as not working if the person was not engaging in any income generating venture; primary occupation was considered as any occupation focusing on extraction of raw materials including all forms of agriculture; secondary occupation referred to the production industry which adds value to raw materials extracted through primary occupation whilst tertiary occupation involved provision of services.

### Analytical approach

First of all, descriptive statistics were conducted whereby prevalence of unintended pregnancy was presented in proportions. This was followed by binary logistic regression since the principal outcome variable was dichotomous. With this, multivariate binary logistic regression analysis was carried out and the results displayed in Table 2. The study employed binary logistic regression because this technique permits extrapolation on a combination of continuous and categorical variables. The principal assumption underpinning the binary logistic regression is that the outcome variable must be dichotomous and the data should not have outliers. The data was weighted with the available sample weight factor within the GDHS dataset in order to subside the effect of sampling bias. However, all missing variables were dropped but were not that much to have any significant effect on the outcome. It is worthy of note that the sample for “decider for contraceptive use” is minimal as a result of the fact that that question was only answered by women who were using contraceptives. The complex design of the survey was also factored whilst STATA version 13 was used for the analysis.

### Ethical consideration

The study involved participation of human subjects, however, since the researcher was not directly involved in the data collection no ethical clearance was sought for this particular study. Meanwhile request for the dataset was made from Measuredhs website. Following this, permission for data usage was granted by Measuredhs after accessing the intent for the request of the dataset.

## Results

### Prevalence of unintended pregnancy

In Table 1, prevalence of unintended pregnancy in Ghana is shown. The overall prevalence of unintended pregnancy stood at 29.8% (data not shown). It was evident from the study that unintended pregnancies were high among 15–19 age cohorts as almost seven out of ten of them had unintended pregnancies (69.4%), which implies that 69.4% of the women interviewed during the survey (aged 15–19) had experienced unintended pregnancies. However, those aged 30–34 had the least share (22.2%). Although a greater share of the women were poor (52.2%), women in the middle wealth status were noted to have relatively high prevalence of unintended pregnancies (39.7%) and similar observation was made among those with secondary/SHS education (36.3%). In addition to the fact that Christians dominated (71.8%), the phenomenon was well pronounced among them (33.3%) than any other religious affiliates.

**Table 1:** Prevalence of unintended pregnancy among Ghanaian women by socio-demographic characteristics (*N* = 4294)

Socio-demographic Characteristics	*N*	Percentage (%)	Unintended Pregnancy *n* (%)
Age cohort			
15–19	183	4.3	126 (69.4)
20–24	740	17.2	313 (42.8)
25–29	1058	24.6	263 (25.1)
30–34	968	22.5	211 (22.2)
35–39	789	18.4	177 (22.8)
40–44	409	9.5	118 (29.5)
45–49	147	3.4	36 (24.8)
Wealth status			
Poor	2241	52.2	620 (28.1)
Middle	812	18.9	312 (39.7)
Rich	1241	28.9	312 (25.6)
Highest education level			
No education	1419	33.1	267 (19.0)
Primary/JHS	869	20.2	284 (33.4)
Secondary/SHS	1837	42.8	658 (36.3)
Higher/tertiary	169	3.9	35 (20.9)
Religion			
Christianity	3085	71.8	1011 (33.3)
Islam	885	20.6	156 (17.7)
Traditionalist	154	3.6	25 (16.4)
No Religion	170	4.0	52 (31.1)
Marital status			
Never married	3041	32.4	231 (63.6)
Married	4243	45.2	595 (21.2)
Cohabiting	1213	12.9	336 (40.5)
Separated	370	3.9	77 (50.0)
Divorced	260	2.8	18 (27.3)
Widowed	269	2.9	13 (16.3)
Residential status			
Rural	2516	58.6	733 (29.5)
Urban	1778	41.4	511 (29.2)
Occupation			
Not working	759	17.7	299 (40.0)
Primary	1316	30.7	313 (24.2)
Secondary	557	13.0	147 (26.9)
Tertiary	1654	38.6	483 (29.7)
Parity			
1–4	1183	27.6	373 (32.1)
4+	3111	72.5	871 (28.4)
Contraceptive use and intention			
Using modern method	1101	25.6	362 (32.9)
Using traditional method	145	3.4	46 (31.7)
Intends to use later	1511	35.2	490 (32.4)
Does not intend to use	1537	35.8	372 (24.2)
Decider for contraceptive use			
Respondent alone	378	26.8	80 (29.3)
Husband alone	151	10.7	25 (24.0)
Joint decision	879	62.3	198 (29.9)
Other	4	0.3	1 (50.0)

Source: Computed from 2014 GDHS dataset

Married women dominated among the respondents (45.2%). The highest prevalence of unintended pregnancy was recorded among women who had never married (63.6%), whilst widows had the least unintended pregnancies (16.3%). When analysed across residential status, the phenomenon was quite high for both rural (29.5%) and urban residents (29.2%). With regard to occupation, it was realised that a significant proportion of the research participants were engaged in tertiary occupation (38.6%) whilst unintended pregnancy was high among non-working women (40.0%). The least prevalence of unintended pregnancy occurred among those engaged in primary occupation (24.2%). The study further revealed that most research participants had more than four children (72.5%), whilst unintended pregnancy was common among those with one to four children (32.1%) as compared to those with more than four children (28.4%). It was again found that no much variation in unintended pregnancies existed between those using modern contraceptives (32.9%), those using traditional method (31.7%) as well as those who intended to use in the future (32.4%). However, the phenomenon was relatively low among women who do not intend to use contraceptive later (24.2%). Women whose husbands make decision on their contraceptive use had the least prevalence of unintended pregnancy (24.0%).

### Correlates of Unintended Pregnancy

Table 2 presents the binary logistic results of correlates of unintended pregnancy. Age, parity and level of education were found to be significantly related with unintended pregnancies. Women in all age categories had less odds of unintended pregnancy as compared to those aged 15–19. Women with higher/tertiary educational qualification were noted to be 2.61 times more likely to have unintended pregnancy than uneducated women (being the reference category) and similar observation was made among those with primary [OR = 1.22, CI = 0.13–0.81] and secondary/SHS education [OR = 1.86, CI = 1.22–2.84].

**Table 2:** Binary Logistic Regression Results on Correlates of Unintended Pregnancy

Variable	OR	95% CI	*P*-value
Age cohort			
15–19	1	[1]	–
20–24	0.71***	[0.09–0.56]	0.000
25–29	0.25***	[0.09–0.70]	0.000
30–34	0.21***	[0.07–0.57]	0.000
35–39	0.16***	[0.06–0.47]	0.000
40–44	0.38***	[0.06–0.53]	0.000
45–49	0.14***	[0.04–0.47]	0.000
Wealth status			
Poor	1	[1]	
Middle	1.42*	[0.48–0.91]	0.008
Rich	0.89**	[0.35–0.79]	0.013
Education level			
No education	1	[1]	
Primary/JHS	1.22***	[0.13–0.81]	0.008
Secondary/SHS	1.86*	[1.22–2.84]	0.031
Higher/tertiary	2.61**	[1.24–5.58]	0.024
Religion			
Christianity	1	[1]	
Islam	0.71***	[0.42–0.99]	0.000
Traditionalist	0.94**	[0.30–0.84]	0.011
No religion	0.70	[0.37–2.19]	0.921
Marital status			
Never married	1	[1]	
Married	0.41**	[0.31–0.91]	0.002
Cohabiting	1.19**	[0.51–0.99]	0.001
Separated	2.14	[1.91–3.81]	0.201
Divorced	1.52***	[0.03–0.79]	0.000
Widowed	1.11	[0.62–2.18]	0.491
Residential status			
Rural	1	[1]	
Urban	1.39	[0.86–1.95]	0.069
Occupation			
Not working	1	[1]	
Primary	1.31**	[0.76–1.11]	0.026
Secondary	1.05	[0.59–1.86]	0.084
Tertiary	1.21	[0.75–1.94]	0.298
Parity			
1–4	1	[1]	
4+	0.25***	[0.16–0.39]	0.000
Contraceptive use and intention			
Using modern method	1	[1]	
Using traditional method	0.82	[0.65–1.06]	0.023
Intends to use later	0.98	[0.83–1.15]	0.019
Does not intend to use	0.65	[0.45–2.77]	0.450
Decision maker for contraceptive use			
Respondent alone	1	[1]	
Husband alone	0.77	[0.45–1.39]	0.031
Joint decision	1.01	[0.41–0.99]	0.011
Other	1.18	[0.06–18.11]	0.821

Source: Computed from 2014 GDHS dataset. *OR* Odds Ratio, *CI* Confidence Interval in square brackets; 1 = reference; * *p* < 0.05, ** *p* < 0.01, *** *p* < 0.001

As compared to Christians, all other religious affiliates were less probable to have unintended pregnancies namely Islam [OR = 0.71, CI = 0.42–0.99], Traditionalists [OR = 0.94, CI = 0.30–0.84] as well as those without any religious affiliation [OR = 0.70, CI = 0.37–2.19]. It was noted that married women had less likelihood of unintended pregnancies as compared to their counterparts who had never married [OR = 0.41, CI = 0.31–0.91]. However, separated women were twice more likely to experience unintended pregnancies as compared with never married women [OR = 2.14, CI = 1.91–3.81]. When the phenomenon was considered across residence, urban women had 1.39 times [CI = 0.86–1.95] likelihood of unintended pregnancies than rural women (reference category). When compared with non-working women, those working in either primary [OR = 1.30, CI = 0.76–1.11], secondary [OR = 1.05, CI = 0.59–1.86] or tertiary [OR = 1.21, CI = 0.75–1.94] sectors were more likely to have unintended pregnancies.

It was additionally revealed that women having more than four children were less probable to have unintended pregnancies [OR = 0.25, CI = 0.16–0.39] as compared to their counterparts with at most four children (being the reference category). On contraceptive use and intention to use, less likelihoods of unintended pregnancies were recorded among traditional method users [OR = 0.82, CI = 0.65–1.06], those intending to use later [OR = 0.98, CI = 0.83–1.15] as well as women who do not intend to use [OR = 0.65, CI = 0.45–2.77] as compared to women using modern contraceptives (reference category). Also, women whose husbands were deciding on contraceptive use were less likely to experience unintended pregnancy [OR = 0.77, CI = 0.45–1.39] than those who were deciding for themselves (reference category), however, in instances where other people were deciding on contraceptive use for the women, likelihood of unintended pregnancy was high as compared to women deciding alone [OR = 1.18, CI = 0.06–18.11].

## Discussion

The study sought to examine the prevalence and correlates of unintended pregnancy in Ghana with data from the 2014 Ghana Demographic and Health Survey. The findings have revealed high prevalence of unintended pregnancy among women within 15–19 age group than women in all other age categories. This is expected due to the fact that most women aged 15–19 in Ghana are more probable to be in Senior High Schools and as a result pregnancies occurring at that time are unexpected. This might further imply their ignorance or limited depth of knowledge about contraceptives and their reproductive system. Unlike older women, these young ones are more likely to engage in unplanned sexual intercourse without any protection possibly due to peer influence or desire for material gains [16]. The higher prevalence of unintended pregnancy among Ghanaian young women is consistent with an earlier observation [17].

High prevalence of unintended pregnancies were found among women who had never married indicating that most pregnancies occurring outside marriage are unintended in Ghana. Pregnancies occurring outside marriage is unacceptable in most Ghanaian societies and as such it is not surprising that most pregnancies occurring out of marriage were unintended [14]. Similarly, Solomon & Mesganaw [18] found a significant relationship between marital status and unintended pregnancy in Ethiopia whilst similar finding has also been reported in Tanzania [19].

It was realised that unintended pregnancy declines as one’s wealth status increases. Unlike the poor, wealthier women are more likely to be highly empowered and have easy access to reproductive health services. This finding corroborates with findings from Iran [20] and other African countries such as the one found at Kenya [21]. Traditionalists, Islam women and those without religious affiliations were less likely to experience unintended pregnancies as compared to Christian women. This might be as a result of doctrinal differences among the women along the lines of religion. This observation is in line with the postulation of the ecosocial theory that a strong linkage exists between women’s thoughts and behaviour about unanticipated pregnancies [11].

The study revealed that urban residents had relatively high tendency of unintended pregnancies. However, no significant relationship existed between residential status and unintended pregnancy and this is consistent with an earlier study [22]. Generally, pregnancies are appreciated and welcome in rural Ghana than urban settings and this might partly account for the high tendency of unintended pregnancy among urban women [23]. Women in urban settings tend to be much focused on economic activities and their holistic development and as such are more probable to declare pregnancies unintended [15].

Those with more than four children were less probable to have unintended pregnancies. This observation might imply that as women have more children they become more conscious about measures to adopt in order to regulate their fertility rather than becoming pregnant unintendedly. This finding contradicts an earlier study by Denise [15]. The differences in outcome might be as a result of variations in other factors operating at these two different study areas as recognised by the ecosocial theory that various social factors induce health in diverse ways.

## Conclusion

Guided by the ecosocial theory, the study has revealed the prevalence of unintended pregnancy in Ghana. The results from this study have highlighted correlates associated with unintended pregnancies in Ghana as age, parity and level of education. This study has contributed substantially to the scanty literature on unintended pregnancy in Ghana by pointing out the prevalence and specific factors contributing to the phenomenon. It has indicated that specific interventions must be targeted at different categories of women. For example, the Reproductive and Child Health unit of the Ghana Health Service in collaboration with non-governmental organisations dedicated to reproductive health ought to intensify access to family planning services among adolescents and young women, women out of marriage more especially separated and divorced women, and the non-working. Expansion in family planning programmes can contribute substantially to reduction in unmet needs for family planning and thereby contribute immensely to combating unintended pregnancies. The study has further indicated that male involvement cannot be overlooked in the quest to curtail unintended pregnancies.

## Abbreviations

DALYs: Disability Adjusted Life Years
GDHS: Ghana Demographic and Health Survey
PHC: Population and Housing Census
